# Evolution and Expression of the Membrane Attack Complex and Perforin Gene Family in the Poaceae

**DOI:** 10.3390/ijms21165736

**Published:** 2020-08-10

**Authors:** Lujun Yu, Di Liu, Shiyi Chen, Yangshuo Dai, Wuxiu Guo, Xue Zhang, Linna Wang, Sirui Ma, Ming Xiao, Hua Qi, Shi Xiao, Qinfang Chen

**Affiliations:** State Key Laboratory of Biocontrol, Guangdong Key Laboratory of Plant Resources, School of Life Sciences, Sun Yat-sen University, Guangzhou 510275, China; liud47@mail2.sysu.edu.cn (D.L.); chenshy77@mail2.sysu.edu.cn (S.C.); daiysh6@mail.sysu.edu.cn (Y.D.); guowx3@mail2.sysu.edu.cn (W.G.); cherxuer@163.com (X.Z.); wangln45@mail2.sysu.edu.cn (L.W.); masr3@mail2.sysu.edu.cn (S.M.); xiaoming3@mail.sysu.edu.cn (M.X.); qihua@mail2.sysu.edu.cn (H.Q.); xiaoshi3@mail.sysu.edu.cn (S.X.)

**Keywords:** MACPF, evolution, expression profile, Poaceae

## Abstract

Membrane Attack Complex and Perforin (MACPF) proteins play crucial roles in plant development and plant responses to environmental stresses. To date, only four *MACPF* genes have been identified in *Arabidopsis thaliana*, and the functions of the *MACPF* gene family members in other plants, especially in important crop plants, such as the Poaceae family, remain largely unknown. In this study, we identified and analyzed 42 *MACPF* genes from six completely sequenced and well annotated species representing the major Poaceae clades. A phylogenetic analysis of *MACPF* genes resolved four groups, characterized by shared motif organizations and gene structures within each group. *MACPF* genes were unevenly distributed along the Poaceae chromosomes. Moreover, segmental duplications and dispersed duplication events may have played significant roles during *MACPF* gene family expansion and functional diversification in the Poaceae. In addition, phylogenomic synteny analysis revealed a high degree of conservation among the Poaceae *MACPF* genes. In particular, Group I, II, and III *MACPF* genes were exposed to strong purifying selection with different evolutionary rates. Temporal and spatial expression analyses suggested that Group III *MACPF* genes were highly expressed relative to the other groups. In addition, most *MACPF* genes were highly expressed in vegetative tissues and up-regulated by several biotic and abiotic stresses. Taken together, these findings provide valuable information for further functional characterization and phenotypic validation of the Poaceae *MACPF* gene family.

## 1. Introduction

The Membrane Attack Complex and Perforin (MACPF) protein family gets its name from a functional domain shared between proteins of the membrane attack complex (MAC) and perforin proteins in a variety of organisms [1]. Proteins of the MACPF family are structurally related to cholesterol-dependent cytolysins [2] and form pores in cellular membranes upon oligomerization [3,4]. MACPF proteins also share structural similarities to proteins of the complement system (C6, C7, C8α, C8β, and C9), which participate in an organism’s immune response to bacterial and viral infections [5].

High resolution X-ray crystallography structure analysis of Perforin, C8α, Plu-MACPF, and GNIP1Aa proteins revealed the mechanism of pore formation by MACPF proteins and the associated disruption of cell membranes [2,6,7,8]. The structures of several MACPF proteins have been resolved, highlighting a conserved core fold for MACPF oligomers, but also variation at the C-terminal domains in vertebrates, including a C2 domain in perforin, a thrombospondin type 1 domain in the complement component C8a, and Epidermal Growth Factor-like domain in complement component C9 [5].

Over the last two decades, several MACPF proteins have been shown to play key roles in animal development and immunity [7,9,10,11]. However, the function and molecular mechanism of MACPF proteins in plants is still poorly understood. To date, only CONSTITUTIVELY ACTIVATED CELL DEATH 1 (CAD1, also named NECROTIC SPOTTED LESIONS 2 or NSL2) and NSL1 in Arabidopsis (*Arabidopsis thaliana*) have been assigned functions in the regulation of the plant immune and salicylic acid (SA)-mediated defense signaling pathways, as well as in the transport of phenolic compounds to restrict fungal invasion [10,12,13,14,15,16].

NSL1 negatively regulates cell death and defense responses in Arabidopsis, as the *nsl1* mutant exhibited retarded growth and developed spotted necrotic lesions on its leaves even in the absence of pathogens [12]. The *nsl1* mutant accumulated high levels of SA, due to the activation of the SA pathway by a Trp-derived secondary metabolite in the mutant background [14]. These results all indicated that NSL1 plays critical roles in the repression of cell death. In addition, CAD1/NSL2 negatively regulated the expression of SA-related defense genes [10,13], and controlled systemic acquired resistance (SAR) by modulation of the hypersensitive response (HR) [16]. Two other Arabidopsis *MACPF* genes have been identified (At1g14780, At4g24290), but their functions remain unclear.

To date, many *MACPF* genes have been discovered in animals and fungi, but only four *MACPF* genes have been identified in Arabidopsis [10], while the functions of the MACPF gene family in other plants, especially in important crop plants such as Poaceae family members, remain largely unknown. The Poaceae family, formerly named Gramineae, comprises many monocotyledonous flowering plants and constitutes the most important source of food in the world, based on both production and dedicated arable land. With advances in sequencing technology, the complete genomes of many Poaceae species have been sequenced and well annotated, including rice (*Oryza sativa*) [17] and purple false brome (*Brachypodium distachyon*) [18], which belong to the BOP (Bambusoideae, Oryzoideae, Pooideae) clade, as well as maize (*Zea mays*) [19], sorghum (*Sorghum bicolor*) [20], foxtail millet (*Setaria italica*) [21], and resurrection grass (*Oropetium thomaeum*) [22] from the PACMAD (Panicoideae, Arundinoideae, Chloridoideae, Micrairoideae, Aristidoideae, Danthonioideae) clade. The completion of these Poaceae genomes opens the door for a systematic analysis of *MACPF* genes on a genomic scale. This systematic analysis will inform functional studies of this gene family across the Poaceae.

Here, we report on the full complement of *MACPF* genes in the Poaceae, with the identification of 42 *MACPF* genes, a description of their expression profiles and the domain organization of their encoded proteins. We categorized plant *MACPF* genes into four classes based on phylogeny, motif organization, and gene structure. We also analyzed the expansion of the *MACPF* gene family across genomes, which revealed that segmental duplications followed by purifying selection were the most important evolutionary driving forces in the Poaceae. Our detailed expression analysis showed that Poaceae *MACPF* genes were preferentially expressed in vegetative tissues, and induced in response to various environmental stresses. Our data provide a foundation for the study of Poaceae *MACPF* gene evolution as well as a useful resource for future functional validation of their roles during plant development and stress responses.

## 2. Results

### 2.1. Identification of MACPF Genes in Poaceae Genomes

We queried 15 selected plant genomes with the Pfam MACPF domain (PF01823) by a combination of Basic Local Alignment Tool for Protein (BLASTP) and HMMER searches. For this analysis, we selected species that represented the major clades of Poaceae and whose genomes were completely sequenced and well annotated. We also included non-Poaceae species for comparison, focusing our efforts on the genomes of four green algae (*Ostreococcus tauri*, *Ostreococcus lucimarinus*, *Chlamydomonas reinhardtii*, and *Volvox carteri*), one moss (*Physcomitrium* [*Physcomitrella*] *patens*), one spikemoss (*Selaginella moellendorffii*), two eudicots (*Arabidopsis thaliana* and grapevine [*Vitis vinifera*]), and six Poaceae (*Oryza sativa*, *Zea mays*, *Sorghum bicolor*, *Brachypodium distachyon*, *Setaria italica*, and *Oropetium thomaeum*; Table 1). We then filtered our candidate genes based on the presence of the MACPF domain, as detected by the Simple Modular Architecture Research Tool (SMART, http://smart.embl-heidelberg.de) and the Conserved Domains Database (CDD, https://www.ncbi.nlm.nih.gov/cdd/). Following this filtering step, we retained 57 sequences as putative *MACPF* genes, in addition to nine partial sequences from the spikemoss *S. moellendorffii* genome (Table 1). We failed to identify a single *MACPF* candidate gene in any of the aquatic green algae tested here. However, we did identify candidate *MACPF* genes in the moss and spikemoss genomes, which suggests that the MACPF protein domain may have appeared after the colonization of land by plants.

We identified six to nine *MACPF* genes in each Poaceae genome: six genes in the resurrection plant, seven in rice, nine in maize, seven in sorghum, six in purple false brome, and seven in foxtail millet (Table 1). We numbered all *MACPF* genes based on their chromosomal location and position (Table S1). Out of 42 Poaceae *MACPF* genes, 16 showed more than two transcript isoforms due to alternative splicing. In these cases, we chose the longest transcripts for further analysis.

The predicted MACPF proteins varied extensively in size, ranging from 406 to 1253 amino acids (Table S1). We determined their physical and chemical properties such as isoelectric point (pI) and molecular weight (MW) using the EXPASY database (http://www.expasy.org/) analysis. pI values varied from 6.38 to 9.48, most being greater than 7.0, indicating that most MACPF proteins are basic. However, resurrection grass OtMACPF5 and foxtail millet SiMACPF6 were acidic, as their pI values were below 7.0 (Table S1). In addition, the MW of MACPF proteins varied widely from 43.2 to 138.3 kDa.

### 2.2. Phylogenetic and Structure Analysis

**Figure 1:**
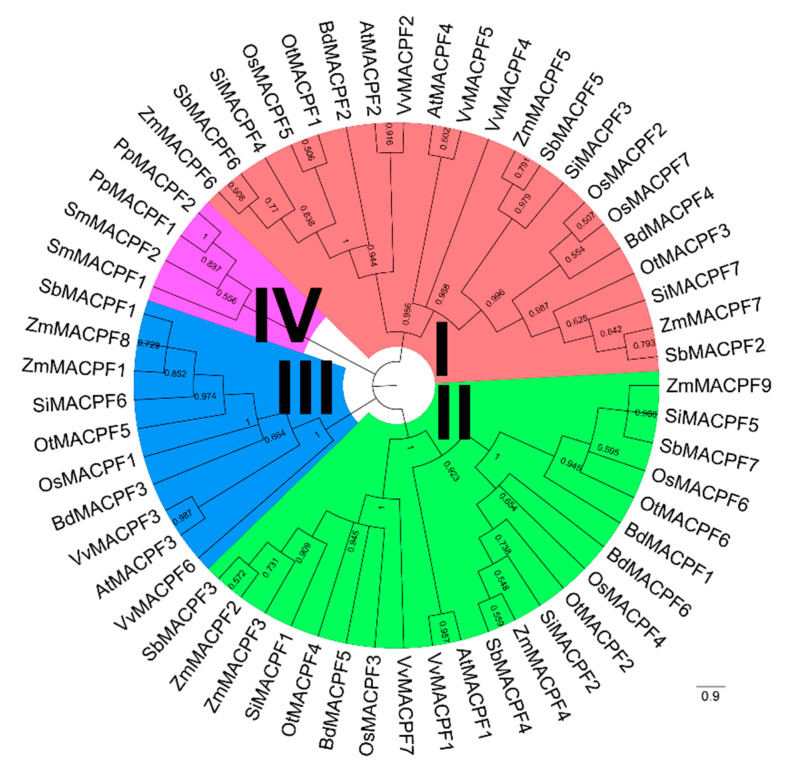
Phylogenetic relationships of 57 plant Membrane Attack Complex and Perforin (MACPF) proteins. We constructed a phylogenetic tree of the *MACPF* family with the MEGA X software package, with Maximum Likelihood method and Jones-Taylor-Thornton (JTT) + gamma distribution evolutionary rates (+G)+I model, using the MACPF catalytic domain encoded by the selected Poaceae genomes. MACPF proteins were classified into four distinct groups, as indicated by the different colors. We visualized the phylogenetic tree with the FigTree software.

To explore the evolution of the plant MACPF family, we constructed a phylogenetic tree with the Maximum Likelihood method and Jones-Taylor-Thornton (JTT) + gamma distribution evolutionary rates (+G)+I model using the sequence of the MACPF domain (Figure 1). Based on the phylogenetic tree and the arrangement of functional domain in individual proteins, we classified the plant MACPF proteins into four groups (Groups I–IV), with bootstrap values of 95, 100, 100, and 55 bootstrap values, respectively (Figure 1). The intra-groups bootstrap values were much higher than the between-group values, supporting this classification. We also used BEAST software to estimate the timing of the divergence in the *MAPCF* gene family. BEAST predicted that Group I diverged the earliest, at an estimated 399.12 million years ago (MYA), and Group III diverged the latest, at 247.12 MYA (Figure S1).

Group IV consisted of two members from moss and two members from spikemoss, suggesting the existence of a bryophyte and lycophyte-specific lineage. The remaining three groups include *MACPF* members from all Poaceae species: Group I had 21 members and Group II had 22 members. Groups I and II therefore comprised the highest number of MACPF proteins, possibly arising from repeated gene duplications (Figure 1). Group I contained two to three *MACPF* members from each Poaceae species, while three to four *MACPF* members were represented per Poaceae species in Group II (Figure 1), suggesting that Group II may have undergone at least one additional round of *MACPF* gene expansion relative to Group I. *MACPF* members belonging to the BOP clade clustered together, as did candidates from the PACMAD clade (Figure 1). Moreover, segmental duplication events were the main cause of the increased number of *MACPF* members. Group I *MACPF* members expanded by dispersed duplication. Aside from two PACPF members in maize, Group III only contained one *MACPF* member from each Poaceae species, suggesting a gene expansion event specific to maize (Figure 1).

**Figure 2:**
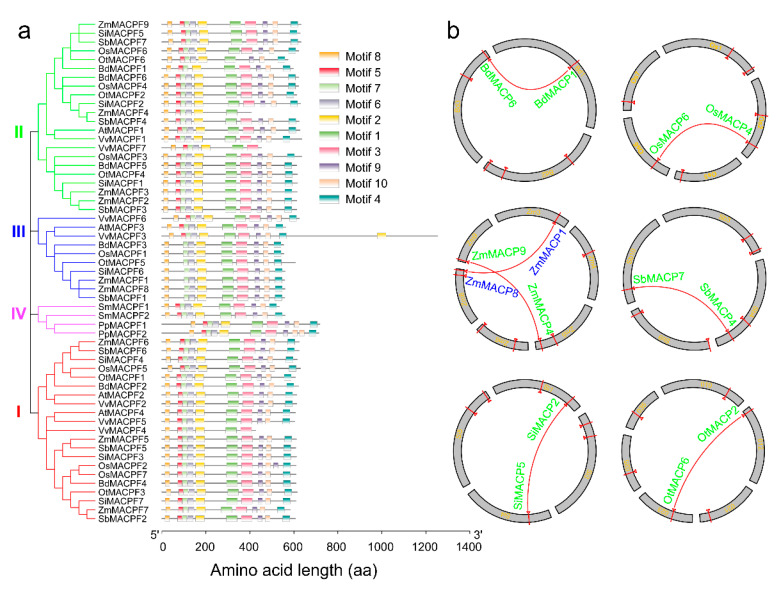
Schematic representation of conserved motifs and segmental duplication analysis. (**a**) Left: phylogenetic tree of the *MACPF* family replotted from Figure 1. Right: conserved motif compositions of plant MACPF proteins, predicted by MEME suite. Each of the 10 conserved motifs are represented by color bars. (**b**) Poaceae *MACPF* genes were mapped to the chromosomes of *Brachypodium distachyon*, *Oryza sativa*, *Zea mays*, *Sorghum bicolor*, *Setaria italica,* and *Oropetium thomaeum*. *MACPF* genes were numbered according to their position on the chromosomes. Segmental duplications of *MACPF* gene pairs are connected by red lines.

The phylogenetic tree largely agreed with the arrangement of 10 protein domains detected in plant MACPF proteins by the multiple EM for motif elicitation (MEME) tool. In addition, these domains shared high E-values, indicating that MACPF proteins were highly conserved across the species considered in this study (Figure 2a and Figure S2). All 57 plant MACPF proteins shared the same nine motifs, which were organized in the same order across all members (Figure 2a and Figure S2), consistent with the high degree of sequence conservation we observed. Interestingly, motif 5 was lost in the Group III Poaceae MACPF proteins, although it was retained in the eudicot Group III proteins (Figure 2a and Figure S2), suggesting a distinct evolutionary trajectory for monocot and eudicot members of Group III.

To better understand the structure of *MACPF* genes, we analyzed the exon–intron organization of the 57 *MACPF* genes using the GSDS website. In agreement with their shared protein motif organizations, most *MACPF* genes exhibited similar exon–intron structures and intron phases, especially within the same group (Figure S3). This analysis also showed that a regulatory untranslated region (5′ or 3′ untranslated region (UTR)) was predicted for 93% (53/57) of the *MACPF* genes, the exception being spikemoss *PpMACPF1* and *PpMACPF2*, resurrection grass *OtMACPF1*, and purple false brome *SiMACPF1* (Figure S3), highlighting the potential for multiple regulatory mechanisms among *MACPF* genes.

### 2.3. MACPF Gene Localization and Gene Duplication

We determined the genomic position of each gene to characterize the pattern of expansion of the *MACPF* gene family. We were able to anchor all 42 *MACPF* genes originating from a Poaceae species to a given chromosome, and observed that *MACPF* genes mapped to three–six chromosomes within each genome (Figure 2b).

Based on criteria for the identification of segmental duplications [23], we documented seven cases of *MACPF* gene pairs that likely arose from duplicated chromosomal segments in the selected Poaceae (Figure 2b). Of those, six *MACPF* pairs belonged to Group II (*BdMACPF1*/*BdMACPF6*, *OsMACPF4*/*OsMACPF6*, *OtMACPF2*/*OtMACPF6*, *SiMACPF2*/*SiMACPF5*, *SbMACPF3*/*SbMACPF7*, and *ZmMACPF1*/*ZmMACPF8*) (Figure 2b). The last segmental duplication pair belonged to Group III (*ZmMACPF4*/*ZmMACPF9*), (Figure 2b). No *MACPF* gene duplications fulfilled the tandem duplication criteria in the Poaceae, suggesting that the *MACPF* gene family expanded by segmental and dispersed duplications, rather than by tandem duplications (Figure 2b).

**Figure 3:**
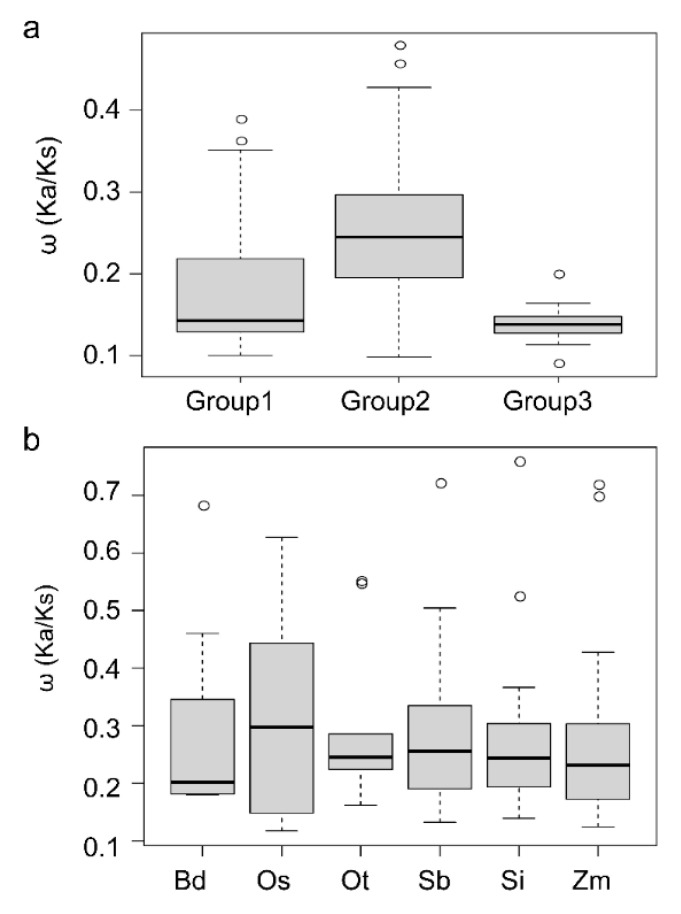
The ω (Ka/Ks ratio) value of *MACPF* genes in Poaceae genomes. Distribution of Ka/Ks values were obtained from pairwise comparisons within Groups I, II and III (**a**), and within each Poaceae genome (**b**). The Y-axis denotes Ka/Ks ratios of *MACPF* genes for each pair. Boxplots were generated in R.

The Ka (non-synonymous distance), Ks (synonymous distance), and ω (Ka/Ks ratio) values are useful metrics to characterize gene evolution and selective pressure [24,25]. In general, values of ω >1, =1, and <1 indicates positive selection, neutral evolution, and purifying selection (also called negative selection) of the selected genes, respectively, according to the neutral theory [26]. To explore the selective pressure and functional diversification of segmental duplication genes, we evaluated ω values for the seven *MACPF* segmental duplicated gene pairs. This analysis revealed that all ω values were below 1 for all gene pairs, and were higher in Group II compared to those of Group III (Table S2), suggesting that segmentally duplicated *MACPF* genes evolved under purifying selection. Indeed, ω values ranged from 0.19 to 0.43 (Table S2) for gene pairs from Group II, compared with 0.15 for the maize gene pair from Group III (Table S2). We also calculated the average ω value of all *MACPF* genes belonging to each group: the mean ω value was 0.17 (Groups I), 0.24 (Group II), and 0.14 (Group III), all well below 1 (Figure 3a). The average Ka/Ks ratio of *MACPF* genes in Group II was the largest (Figure 3a), implying that Group II experienced more relaxation of purification than the other two groups. In addition, the Ka/Ks ratio was the smallest in Group III, suggesting a much stronger susceptibility to functional differentiation in Group III. We detected no positive selection among *MACPF* genes. The above results therefore implied that MACPF genes belonging to different groups faced varying degrees of purifying selection pressure during the expansion of this gene family.

We next determined the average ω values of *MACPF* paralogous pairs in each Poaceae species: they ranged from 0.27 to 0.31 and were therefore all less than 1 (Figure 3b), implying again that purifying selection may be the most important evolutionary driving force during *MACPF* gene expansion in the Poaceae. Purple false brome and maize had the smallest Ka/Ks ratios, indicating that maize *MACPF* genes were much more susceptible to functional differentiation during evolution. Rice showed the largest average Ka/Ks ratio (Figure 3b), suggesting that rice *MACPF* genes experienced more relaxation of purification than other five Poaceae. These results suggested similar selection forces in play between groups as well as within each Poaceae genome.

### 2.4. Synteny Analysis of the MACPF Gene Family in Poaceae

To examine the origin and evolutionary history of *MACPF* genes, we used the MCScanX software to determine the degree of synteny and collinearity relationship of *MACPF* genes across the Poaceae. Accordingly, we calculated synteny blocks for *MACPF* genes with MCScanX, setting the parameter of the e-value to <1 × 10^−10^ [27], followed by graphical representation using the TBtools software package [28].

**Figure 4:**
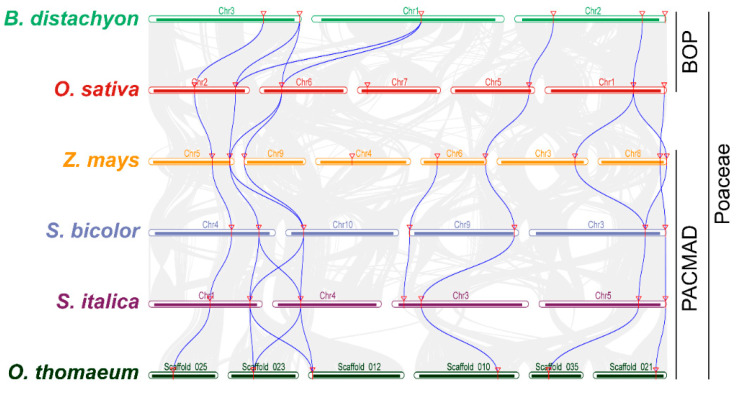
Chromosomal synteny and collinearity analysis of *MACPF* genes across the Poaceae. Putative orthologous genes among Poaceae genomes are connected by lines using the MCScanX software. Red triangles represent *MACPF* genes in *Brachypodium distachyon*, *Oryza sativa*, *Zea mays*, *Sorghum bicolor*, *Setaria italica,* and *Oropetium thomaeum*. Blue solid lines represent collinearity relationships of orthologous *MACPF* gene pairs.

We identified 40 orthologous *MACPF* gene pairs in Poaceae genomes, including seven blocks between purple false brome and rice, seven blocks between rice and maize, nine blocks between maize and sorghum, nine blocks between sorghum and foxtail millet, and eight blocks between foxtail millet and resurrection grass (Figure 4). The comparable number of *MACPF* orthologous gene pairs between Poaceae genomes is indicative of the higher level of evolutionary relationship of *MACPF* gene family members in the Poaceae. We only detected one orthologous gene pair (*ZmMACPF6*/*SbMACPF4*/*SiMACPF4*) shared across maize, sorghum and foxtail millet (Figure 4), suggesting a special *MACPF* evolution within the PACMAD clade.

### 2.5. Analysis of Stress-Responsive cis-Regulatory Elements in MACPF Promoters

**Figure 5:**
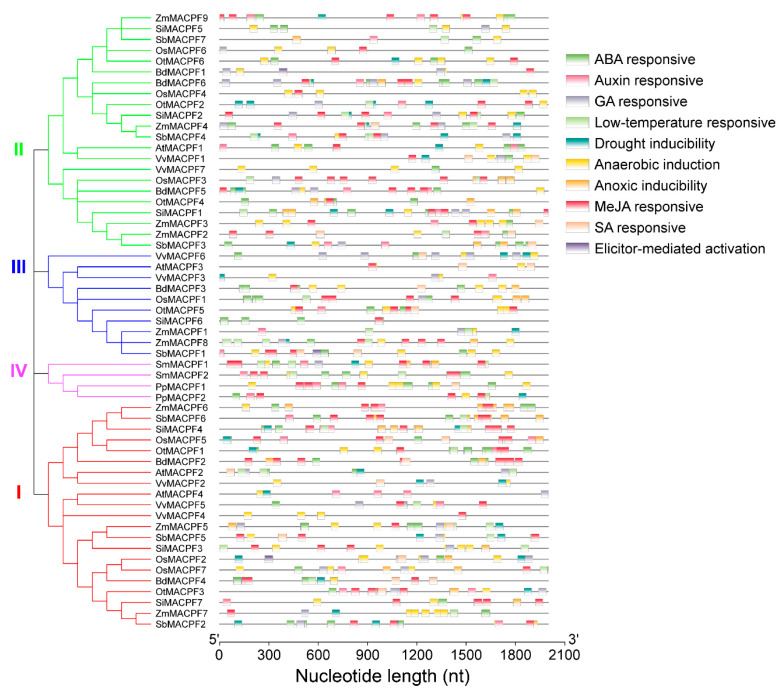
Promoter analysis of *MACPF* genes in the Poaceae. Left: phylogenetic tree of the *MACPF* family, replotted from Figure 1. Right: regulatory elements of the *MACPF* promoters, as predicted by the PlantCARE database. *cis*-regulatory elements in the promoter regions are indicated as different colored-blocks.

*MACPF* genes play important roles in defense response in plants and animals [2,14]. To discover *cis*-regulatory elements (CREs) relevant to stress responses such as abiotic and biotic stresses, we subjected 2 kb of sequence upstream of each Poaceae *MACPF* gene to analysis through the PlantCARE database. We identified 10 distinct *cis*-elements distributed over the length of each promoter region, classified into phytohormone-responsive (five elements), plant defense response-related, drought- or cold-responsive, related to anaerobic or anoxic stress (Figure 5). Our analysis did not include light-responsive elements in *MACPF* promoters, which was usually not associated with stress response. We identified 834 potential *cis*-regulatory elements across the 57 *MACPF* promoters (Figure 5). These elements included 161 putative ABREs (ABA-responsive elements) in 46 *MACPF* promoters (Figure 5), where they play a role in responses to salt and osmotic stress [29]. In addition, the promoters of 31 *MACPF* genes contained 48 putative MBSs (MYB binding sites), mediating drought stress responses, and the promoters of 28 *MACPF* genes contained 46 putative LTREs (low temperature responsive elements) (Figure 5), involved in responses to cold stress. Other putative motifs included 53 GARE (gibberellin-responsive) motifs in 32 *MACPF* promoters, and 167 putative anaerobic- or anoxic-related motifs (ARE and GC-motif), associated with anaerobic or low oxygen stress, in the promoters of 55 *MACPF* genes (Figure 5), suggesting that a subset of *MACPF* genes might be involved in anoxia responses.

In addition, we identified several biotic stress-related elements, such as AT-rich motif (TAAAATACT) in *OsMACPF2*, *BdMACPF1,* and *SbMACPF1* (Figure 5), responsible for elicitor-mediated activation of plant defenses. The promoters of 47 *MACPF* genes contained 282 putative TGACG or CGTCA motifs responsible for methyl jasmonate (MeJA) responses (Figure 5). Finally, we found 26 putative TCA-element or SARE (SA-Responsive Element) motifs involved in SA responses in the promoters of 20 *MACPF* genes (Figure 5). These results strongly suggested that plant *MACPF* genes are likely involved in multiple stress responses.

### 2.6. Expression Profile of MACPF Genes in Poaceae

**Figure 6:**
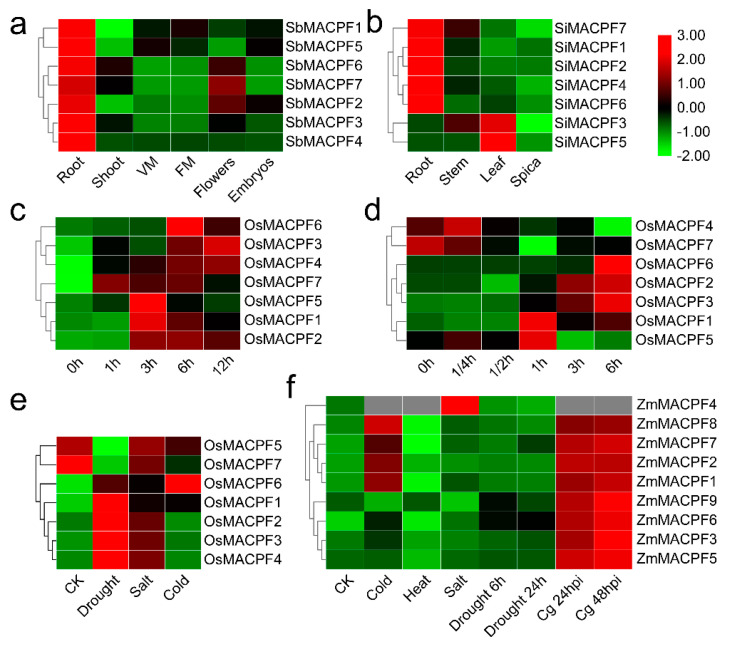
Expression patterns of *MACPF* genes in development and response to stress. Tissue-specific expression patterns of *MACPF* genes in sorghum (**a**) and foxtail millet (**b**), were obtained from previously published data. Red and green colors represent higher and lower expression, respectively. Sorghum samples: root, shoot, VM (vegetative meristem) of the vegetative tissues, and FM (floral meristem), flowers, embryos of reproductive tissues. Foxtail millet samples: root, stem, leaf of the vegetative tissues, and spica (tassel) of the reproductive tissues. Expression profile of *MACPF* genes under abiotic and biotic treatments were obtained from previously published data. Expression profile of *OsMACPF* genes under methyl jasmonate (MeJA) treatment in rice shoots (**c**) and roots (**d**). (**e**) Expression profile of *OsMACPF* genes during drought, salt and cold stress. (**f**) Expression profile of *ZmMACPF* genes during cold, heat, salt, drought stress, and *Colletotrichum graminicola* (*Cg*) infection. Heatmaps were drawn in R. Red and green colors represent higher and lower expression genes in different tissues or exposed to the stresses, respectively.

To explore the expression patterns of Poaceae *MACPF* genes across diverse developmental tissues and organs, we collected previously published RNA-Seq and microarray datasets available from the Gene Expression Omnibus (GEO) database, followed by analysis as previously described [30]. An examination of *MACPF* tissue expression patterns showed that genes belonging to Group III displayed higher transcript levels relative to genes from Group I and Group II in the Poaceae species purple false brome, rice, sorghum, foxtail millet, and maize (Figure 6a,b, and Figures S4–S6). In addition, most *MACPF* genes were highly expressed in vegetative tissues (root, shoot, leaf, stem, or node), when compared to reproductive tissues (inflorescence, flower, or seed) in the same Poaceae species (Figure 6 and Figures S4–S6), suggesting that *MACPF* genes may play important roles in vegetative organs and tissues. Further investigation indicated that rice *OsMACPF2/5/7* in Group I, and *OsMACPF3/4/6* in Group II (Figure S4a), as well as the segmental duplication pair *BdMACPF1* and *BdMACPF6* in purple false brome, were differently expressed in the selected samples (Figure S5), indicating functional differentiation within the *OsMACPF* groups. The foxtail millet genes *SiMACPF4* and *SiMACPF7* showed similar expression profiles, which were different from that of *SiMACPF3* in Group I (Figure 6a), suggesting potential functional redundancy and differentiation inside the *SiMACPF* genes.

Sorghum *SbMACPF2/5/6* in Group I, and *SbMACPF3/4/7* in Group II were all expressed at high levels in the root (Figure 6b), suggestive of their potential functional redundancy. Maize *ZmMACPF1* and *ZmMACPF8*, corresponding to a segmental duplication pair from Group III, similarly showed a similar expression profile, raising the possibility of their functional redundancy (Figure S6), while the maize *ZmMACPF4* and *ZmMACPF9* segmental duplication pair from Group II (Figure S6) showed distinct expression pattern, indicative of functional differentiation. Collectively, five segmental duplication pairs from *MACPF* Group II, showed the different expression pattern consistent with functional differentiation.

### 2.7. Involvement of MACPF Genes in Response to Stresses

Plants are continuously challenged by a multitude of stresses, including abiotic and biotic stimuli. Two Arabidopsis *MACPF* genes function in plant responses to stresses [10,12,13,15]. To determine whether *MACPF* genes were involved in stresses, we analyzed the expression pattern of Poaceae *MACPF* genes upon abiotic or biotic treatments. We explored publicly available data in the Gene Expression Omnibus (GEO) database [31,32,33,34,35], which compiles Poaceae gene expression data across multiple experiments. We observed that all seven *OsMACPF* genes were up-regulated upon jasmonic acid (JA) treatment in the shoots or roots (Figure 6c,d). In sorghum, *SbMACPF7* was induced by abscisic acid in the shoot, (Figure S4b) and by high pH in the root (Figure S4c), respectively. As in sorghum, drought induced the expression of the rice genes *OsMACPF1/2/3/4/6*, and salt treatment increased transcript levels for the rice genes *OsMACPF1/2/3*, and the rice gene *OsMACPF6* was itself induced by cold stimulus (Figure 6e). In the case of maize, nine *ZmMACPF* genes were induced by abiotic or biotic stresses (Figure 6f). *ZmMACPF1/2/7/8* were up-regulated by cold stress, while *ZmMACPF4* and *ZmMACPF6* were induced during salt or drought stress, respectively (Figure 6f). Eight *ZmMACPF* genes were also highly induced at 24 and 48 h following inoculation with the fungus *Colletotrichum graminicola* (Figure 6f). These results suggest that Poaceae *MACPF* genes are involved in plant responses to abiotic and biotic stresses. The segmental duplication pairs *OsMACPF4/OsMACPF6*, *SbMACPF3/SbMACPF7,* and *ZmMACPF4/ZmMACPF9* from Group II (Figure 6c–e and Figure S4b,c) showed distinct expression profiles upon exposure to various stresses, suggesting their functional differentiation. By contrast, the segmental duplication pair *ZmMACPF1/ZmMACPF8* from Group III showed similar regulation after exposure to stress (Figure 6f), indicating their potential functional redundancy.

### 2.8. Expression of Rice MACPF Genes in Response to Jasmonic Acid Treatment

**Figure 7:**
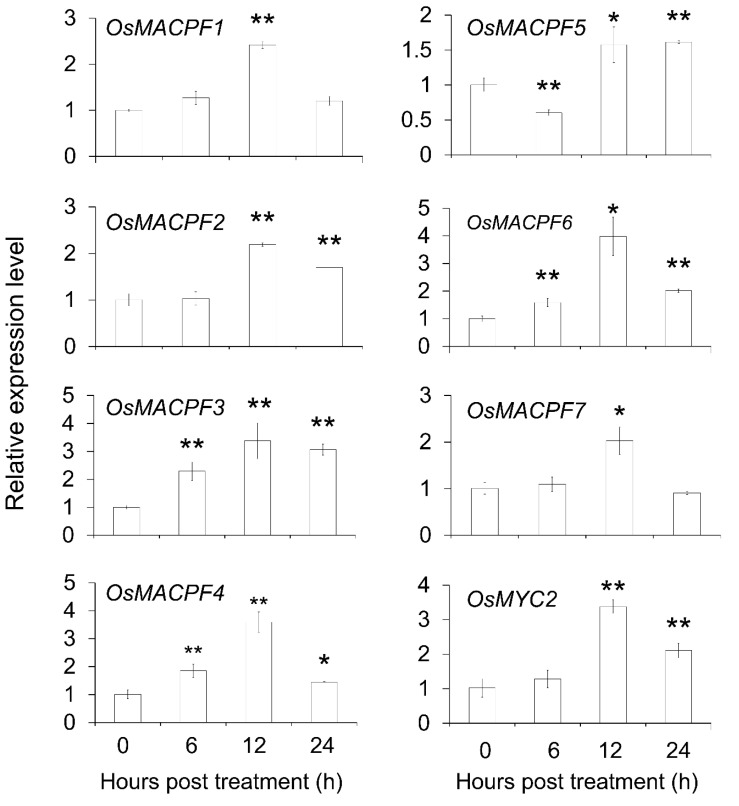
Expression profile of *OsMACPF* genes upon MeJA treatment. Quantitative reverse transcription PCR (qRT-PCR) analysis of *OsMACPF1-7* genes after JA treatment for 6, 12, and 24 h. Rice plants at the four-leaf stage were sprayed with 100 μM MeJA. They were placed in a greenhouse 28/23 °C (light/dark), light period 10 h light/14 h darkness. *OsMYC2* was the positive control for MeJA treatment. Transcript levels were normalized using *OsACTIN* as the internal reference. Each data point represents the average of three biological repeats. * *p* < 0.05; ** *p* < 0.01 by Student’s *t*-test.

To further investigate the function of rice *MACPF* genes upon stress, we sprayed wild type Nipponbare rice plants with 100 μM MeJA. We collected leaves at 6, 12, and 24 h after treatment and extracted total RNA, which we then used as starting material for reverse-transcription and quantitative PCR (qRT-PCR) analysis of seven *OsMACPF* genes. Their transcript levels rose and peaked after 12 h. The marker gene *OsMYC2* in the JA signaling pathway was induced over three-fold after the 12 h JA treatment (Figure 7). Similarly, the rice *OsMACPF1/2/3/4/6/7* saw an increase in their transcript levels larger than two-fold upon MeJA treatment for 12 h (Figure 7), indicating that most *OsMACPF* were highly induced by JA treatment, and may therefore participate in plant responses to JA treatment.

## 3. Discussion

MACPF proteins perform a number of functions over the course of an organism’s development, as well as within multiple signaling pathways and in response to pathogen attacks in fungi and mammals [36,37,38]. To date, only the Arabidopsis *MACPF* gene family has been characterized in some detail [10]. Similar genomic information on the *MACPF* gene family is lacking in other plant species, especially in members of the Poaceae. Here, based on phylogenetic analysis, domain organization, and gene structure analysis, we combined evolution and expression profile analysis to explore the evolution and function of the *MACPF* family, which expands our understanding of MACPF proteins in the Poaceae.

### 3.1. The MACPF Gene Family is Conserved across Land Plants

Only a subset of Arabidopsis *MACPF* genes have been described and assigned a biological function [10]. By combining BLASTP searches with HMMER model analysis, we identified a total of 57 *MACPF* genes in 14 selected plant genomes, among which 42 mapped to Poaceae genomes. We failed to identify *MACPF* genes in green algae. The first occurrence of a *MACPF* gene was detected in the moss *P. patens* (Table 1), suggesting that the *MACPF* gene family evolved after the colonization of land by plants, making them a land plant-specific gene family.

*MACPF* genes varied from six to nine among the selected Poaceae, with no correlation between *MACPF* gene number and genome size (Table 1). For instance, rice and foxtail millet both contained seven *MACPF* genes, although the foxtail millet genome is roughly one third bigger than that of rice. The maize genome, with its 2.4 Gb size, is thought to have undergone one additional whole genome duplication event compared to other members of the Poaceae [39]. Accordingly, the maize genome contained the highest number of *MACPF* members, with two segmental duplication pairs (Figure 2b). In addition, there were comparable numbers of genes in Groups I, II, and III, indicating that the *MACPF* gene family may have followed very similar evolutionary directions in the Poaceae, with the exception of maize, which was also supported by its Ka/Ks ratio (Figure 3). More importantly, the high and consistent conservation of *MACPF* genes in the Poaceae is strongly supported by their similar gene lengths, their exon–intron structures, the deduced amino acid sequences of the proteins they encode, as well as their physical and chemical properties (pI and MW) and their functional motif organizations (Figure 1, Figure 2a, and Figure S3). These results indicate that *MACPF* family members are highly evolutionarily conserved. We hypothesize that they are essential for specific roles in development and during disease resistance across Poaceae species. The conserved motifs arrangement of functional domains among the *MACPF* groups indicates that the protein architecture is highly conserved, although their associated functions largely remain to be elucidated.

Alternative splicing has the potential to increase protein functional diversity for plant development and response to stresses [40,41]. Out of 42 Poaceae *MACPF* genes, 16 showed more than two gene models caused by alternative splicing, with the potential to diversify protein function in the face of stress conditions. Untranslated regions (UTRs) have been suggested to play important roles during the control of gene expression and mRNA translation, including efficiency and stability [42,43]. Our gene structure analysis showed that 90.4% (38/42) of Poaceae *MACPF* genes contained a 5′ or 3′ UTR, with the exception of *SmMACPF1*, *SmMACPF2*, *OtMACPF1*, and *BdMACPF1* (Figure S3), indicating a wide range of potential mechanisms regulating *MACPF* genes. The results above suggest that the diversity of transcripts caused by alternative splicing and the presence/absence of 5′ and 3′ UTRs might increase protein diversity of the Poaceae *MACPF* gene family.

### 3.2. MACPF Duplications are an Important Feature Across the Poaceae

The 42 *MACPF* genes from Poaceae species were unevenly distributed across the Poaceae chromosomes, which is consistent with an ancient aneuploidy event in rice [44], in contrast to a whole genome duplication or polyploidization event. The chromosomal locations of *MACPF* genes indicated that *MACPF* genes largely do not cluster in Poaceae genomes (Figure 2b). An analysis of synteny and collinearity within and between Poaceae genomes revealed that *MACPF* genes expanded by segmental duplication, resulting in the scattered occurrence of *MACPF* genes across the genome [23]. Furthermore, we failed to detect tandem duplications, implying that segmental duplication played vital roles in *MACPF* gene family expansion. Segmental duplicated *MACPF* gene pairs belonged to the same group, which is consistent with previously identified duplication events in the Poaceae [45]. Paralogous *MACPF* genes, originating from segmental duplications, also showed similar exon–intron structures and belonged to the same subfamilies, with high bootstrapping values in the phylogenic analysis (Figure 1, Figure 2a, and Figure S3), indicating that there was no other domain addition or deletion during their evolution. Poaceae *MACPF* genes in Group I, II, and III, all had ω values below 1, suggesting that purifying selection was the main evolutionary driving force during *MACPF* gene family expansion in the Poaceae, as also described in other gene families [46].

Duplicated genes may experience sub-functionalization, neo-functionalization, or non-functionalization after gene duplication [47]. The maize segmental duplication pair *ZmMACPF1* and *ZmMACPF8*, within Group III, displayed a similar expression profile (Figure S6), indicating potential functional redundancy. Six Poaceae segmental duplication gene pairs in Group II showed distinct expression patterns between duplicated *MACPF* genes (Figure 6 and Figures S4–S6), suggesting divergence and sub-functionalization of segmental duplicated genes during *MACPF* gene family expansion in the Poaceae.

### 3.3. MACPF Function and Gene Expression

When confronted with environmental stresses, plants initiate numerous physiological, biochemical, molecular, and cellular changes to acclimatize to the stress, including changes in gene expression [48]. Several *MACPF* genes have been reported to be involved in plant development and stress responses [10,12,15]. A re-analysis of available RNA-seq or microarray data provided support for these claims (Figure 6 and Figures S4–S6). Indeed, *MACPF* genes were preferentially expressed in vegetative tissues, suggesting that *MACPF* genes might play an important role at this developmental stage.

In this study, we identified 834 *cis*-regulatory elements within the promoters of *MACPF* genes (defined as the 2 kb upstream of the ATG), many representing adaptation-related elements, for example phytohormone- and stress-related motifs, indicating that *MACPF* genes may play critical roles in plant responses to environmental adaptations or within phytohormone signaling pathways, including abscisic acid, auxin, gibberellin, MeJA, and salicylic acid. Stress and phytohormone responses are strongly connected [43,49], which was reflected by our promoter analysis. Phytohormone or stress-related *cis*-regulatory elements were predominant across all promoters, indicating that *MACPF* genes may play important roles in plant response to stress.

Gene expression patterns in developmental stages and stress responses provided useful clues for gene function. Generally, Poaceae *MACPF* genes displayed different overlapping expression pattern in the selected samples (Figure 6 and Figure S4), suggesting that *MACPF* genes may play diverse and complex functions during plant development and responses to environmental stress in the Poaceae. Our experiment illustrated the strong induction of six *OsMACPF* genes upon MeJA treatment, which is consistent with the numerous MeJA-responsive elements identified in their promoters (Figure 5) as well as with the RiceXPro database (Figure 6c), suggesting that *OsMACPF* genes may take part in plant responses to environmental stimuli.

## 4. Materials and Methods

### 4.1. Data Retrieval of MACPF Genes

To uncover the entire *MACPF* gene family in Poaceae, we used four Arabidopsis *MACPF* genes [10] as query with the Basic Local Alignment Tool for Protein (BLASTP) method against sequenced representative genomes, with the E-value cutoff of 10^−5^. We searched the genomes of four chlorophytes (green algae, *Ostreococcus tauri*, *Ostreococcus lucimarinus* [50], *Chlamydomonas reinhardtii* [51], and *Volvox carteri* [52]), bryophyte moss (*Physcomitrium* [*Physcomitrella*] *patens*) [53], spikemoss (*Selaginella moellendorffii*) [54], eudicots *Arabidopsis thaliana,* and *Vitis vinifera* [55]. We also performed the same search against the genomes of monocots with completely sequenced and well annotated genomes, belonging to the BOP (Bambusoideae, Oryzoideae, Pooideae) clade within the Poaceae, which includes *Oryza sativa* [17] and *Brachypodium distachyon* [18], and the PACMAD (Panicoideae, Arundinoideae, Chloridoideae, Micrairoideae, Aristidoideae, Danthonioideae) clade, including *Zea mays* [19], *Sorghum bicolor* [20], *Setaria italica* [21], and *Oropetium thomaeum* [22]. In addition, we downloaded the corresponding Pfam entry (PF01823) for the MAC/Perforin domain from the Pfam database [56] and used it as a seed to identify MACPF candidates in the selected genomes listed above, as well as in the publicly available Ensembl Plants [57] and Phytozome databases [58], with the aid of the HMMER 3.0 software [59], using the E-value cutoff of 10^−5^, as described previously [60]. We further examined candidate MACPF proteins thus identified for the presence of a complete MACPF domain by running them through the SMART [61], CDD [62], InterProscan [63], and Pfam [56] databases, which look for conserved functional domains.

### 4.2. Gene Exon–Intron Structure Predictions

We determined the predicted exon–intron organization (structure) by using the Gene Structure Display Server (GSDS 2.0) [64], and illustrated the results with TBtools [28].

### 4.3. Analysis of Domain Combinations and Architecture of MACPF Promoters and MACPF Proteins

We employed the protein databases SMART [61], InterProscan [63] and MEME (multiple EM for motif elicitation) [65] to characterize the complement of functional domains detected for all individual MACPF proteins. In addition, we retrieved *MACPF* promoters, defined as the 2000 bp upstream of the respective coding regions, and submitted them to the PlantCARE database [66] (http://bioinformatics.psb.ugent.be/webtools/plantcare/html) to identify putative *cis*-regulatory elements.

### 4.4. Phylogenetic Analysis

We performed an alignment of the catalytic domains of all MACPF proteins with ClustalX 2.0 [67] using the following parameters: BLOSUM 30 for the protein weight matrix, and gap extension penalty set to the default. We then constructed the corresponding phylogenetic tree of MACPF catalytic domains using MEGA X [68] with the maximum likelihood method (ML). The ML method included parameters from the Jones-Taylor-Thornton (JTT) model and Gamma distribution evolutionary rates (+G), which was considered as the most suitable substitution pattern estimated by maximum likelihood evaluation of 56 different amino acid substitution models (Table S3), pairwise deletions and 1000 bootstraps to estimate branch support. We used the FigTree software (http://tree.bio.ed.ac.uk/software/figtree/) to visualize the phylogenic tree. In addition, we used BEAST [69] to date the internal nodes of the plant MACPF phylogenetic tree.

### 4.5. Duplication Events and Synteny Analysis of MACPF Genes in the Poaceae

We used MCScanX [27] to identify intragenomic (evidence of whole-genome duplications) and intergenomic (between related species) syntenic blocks within the Poaceae, using default parameters. We also conducted a segmental duplication analysis of all *MACPF* genes, by expanding the analysis to the 10 genes flanking each *MACPF* gene [23]. We visualized and illustrated the results with the Tbtools software package [28], which connects segmental duplication genes by links.

### 4.6. Gene Expression Analysis

We explored the tissue- and stress-specific expression pattern of *MACPF* genes in the Poaceae by re-analyzing previously published deep sequencing of the transcriptome (RNA-seq) or microarray expression datasets for rice (GSE6901) [31], sorghum (GSE50464) [32], foxtail millet (GSE36391) [33], maize (bar.utoronto.ca/efp_maize) [34], and purple false brome (E-MTAB-5491) [35]. We selected differentially expressed genes with an absolute fold-change of at least 2 relative to the control samples and used the R software to cluster gene expression values based on Z scores as previously described [30].

### 4.7. RNA Extraction and Quantitative RT-PCR

Rice seedlings for the cultivar Nipponbare, grown in a greenhouse under 28/23 °C (day/night), and a photoperiod of 10-h light/14-h darkness, were sprayed at the four-leaf stage with 100 μM MeJA (Sigma-Aldrich, St. Louis, MO, USA) and then the seedlings were collected for RNA extraction after 6, 12, and 24 h. We performed qRT-PCR (quantitative reverse transcription PCR) as described [70,71]. Primers used in this study are listed in Table S4. *OsACTIN* was selected as the internal control, with *OsMYC2* as the MeJA response marker gene. 

## 5. Conclusions

Here, we characterized the full complement of *MACPF* genes in six representative Poaceae genomes, for a total of 42 *MACPF* genes showing uneven distribution across the chromosomes. The plant *MACPF* genes clustered into four groups based on phylogenetic data, domain organization, motif composition, and gene structure. Intra-genome synteny analysis indicated that segmental duplication played important roles during *MACPF* gene family expansion. Inter-genome collinearity analysis revealed 40 *MACPF* orthologous gene pairs. ω value calculation indicated that the most important evolutionary driving force of the *MACPF* gene family expansion was purifying selection in Poaceae. Our comprehensive analysis revealed that several Poaceae *MACPF* genes may play significant roles during plant vegetative growth and environmental stresses adaptation. These results provide new understanding of *MACPF* gene evolution, and constitute a useful resource for further functional characterization of Poaceae *MACPF* genes during development and stress adaptation.

## Figures and Tables

**Table 1:** Numbers of *MACPF* genes in selected plants.

Plant	Clade	Genome Size (Mb)	Total	Group I	Group II	Group III	Group IV
Green alga (*Ostreococcus tauri*)	Chlorophytes	12.56	0	0	0	0	0
Green alga (*Ostreococcus lucimarinus*)	Chlorophytes	13.2	0	0	0	0	0
Green alga (*Chlamydomonas reinhardtii*)	Chlorophytes	120	0	0	0	0	0
Green alga (*Volvox carteri*)	Chlorophytes	138	0	0	0	0	0
Moss (*Physcomitrium* [*Physcomitrella*] *patens*)	Bryophytes	472	2	0	0	0	2
Spikemoss (*Selaginella moellendorffii*)	Lycophytes	100	2	0	0	0	2
Eudicot (*Arabidopsis thaliana*)	Eudicots	135	4	2	1	1	0
Eudicot (*Vitis vinifera*)	Eudicots	500	7	3	2	2	0
Monocot (*Brachypodium distachyon*)	Monocots	355	6	2	3	1	0
Monocot (*Oryza sativa*)	Monocots	370	7	3	3	1	0
Monocot (*Zea mays*)	Monocots	2400	9	3	4	2	0
Monocot (*Sorghum bicolor*)	Monocots	730	7	3	3	1	0
Monocot (*Setaria italica*)	Monocots	490	7	3	3	1	0
Monocot (*Oropetium thomaeum*)	Monocots	245	6	2	3	1	0
Total	-	-	57	21	22	10	4

## Supplementary Materials

Supplementary materials can be found at https://www.mdpi.com/1422-0067/21/16/5736/s1.

## Abbreviations

ABA	Abscisic acid
ABRE	ABA-responsive element
AS	Alternative splicing
Bd	*Brachypodium distachyon*
CAD1	Constitutively activated cell death 1
GA	Gibberellin
GSDS	Gene Structure Display Server
HMM	Hidden Markov model
Ka	Non-synonymous distance
Ks	Synonymous distance
LTRE	Low temperature responsive element
MAC	Membrane attack complex
MACPF	Membrane Attack Complex and Perforin
MBS	MYB binding site
MeJA	Methyl jasmonate
MEME	Multiple EM for motif elicitation
MW	Molecular weight
NSL1	Necrotic spotted lesions 1
NSL2	Necrotic spotted lesions 2
Os	*Oryza sativa*
Ot	*Oropetium thomaeum*
pI	Isoelectric point
Pp	*Physcomitrium* (*Physcomitrella*) *patens*
qRT-PCR	Quantitative reverse transcription PCR
SA	Salicylic acid
Sb	*Sorghum bicolor*
Si	*Setaria italica*
Sm	*Selaginella moellendorffii*
Zm	*Zea mays*
