# Supplementary material for: Evolution and Expression of the Membrane Attack Complex and Perforin Gene Family in the Poaceae
Source: Int J Mol Sci. 2020 Aug 10;21(16):5736. doi: 10.3390/ijms21165736 (PMC7460961; doi:10.3390/ijms21165736)
Supplement: Supplementary file 1 [file ijms-21-05736-s001.zip › Poaceae MACPF Supplemental Data Revision with Track Changes.docx]

Supplemental Data

Evolution and Expression of the Membrane Attack Complex and Perforin Gene Family in the Poaceae


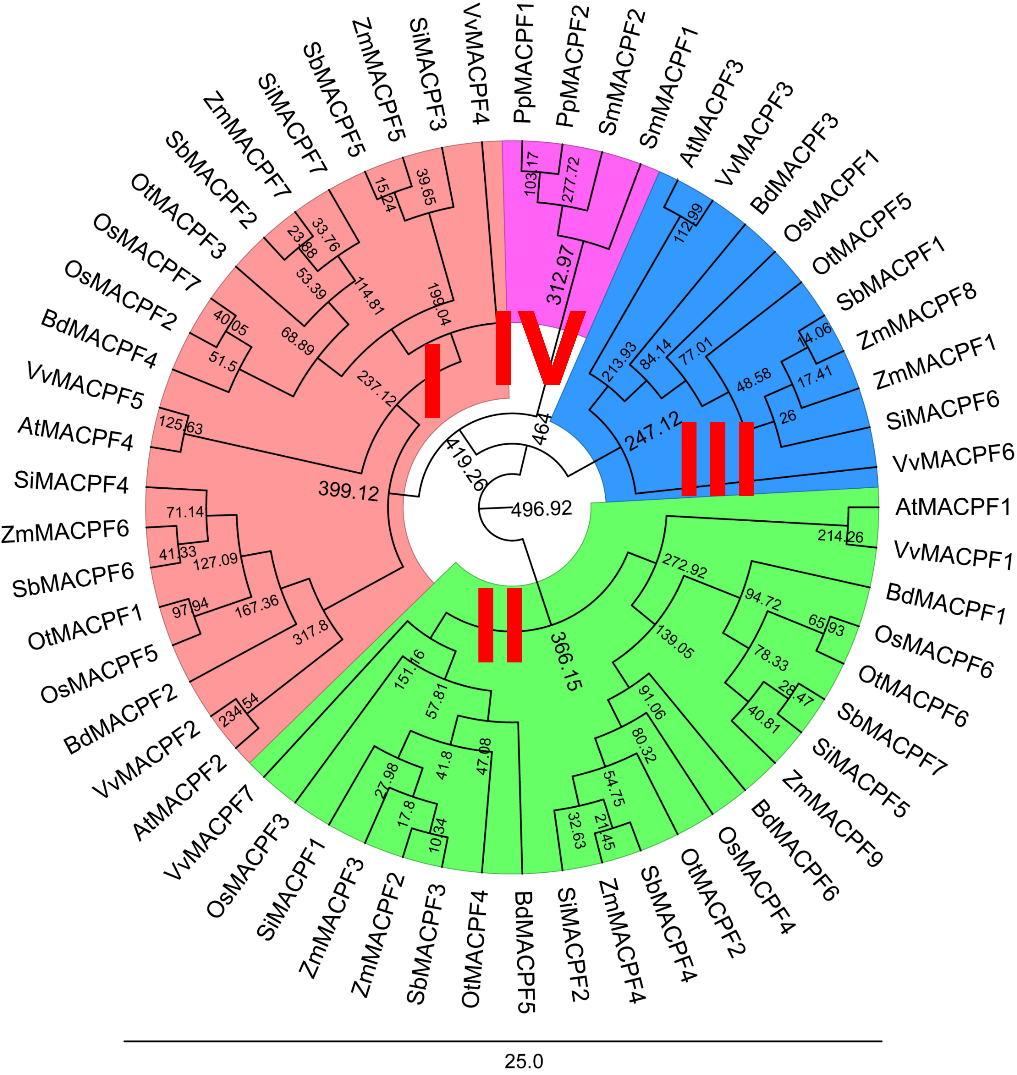


**Figure S1.** The Bayesian phylogenetic tree analysis was reconstructed by employing BEAST software. BEAST version 2.5.2 software was used to dating the internal nodes of the plant MACPF phylogenetic tree. The pairwise divergence time was set as median time 480 MYA, according the TimeTree web (http://www.timetree.org).


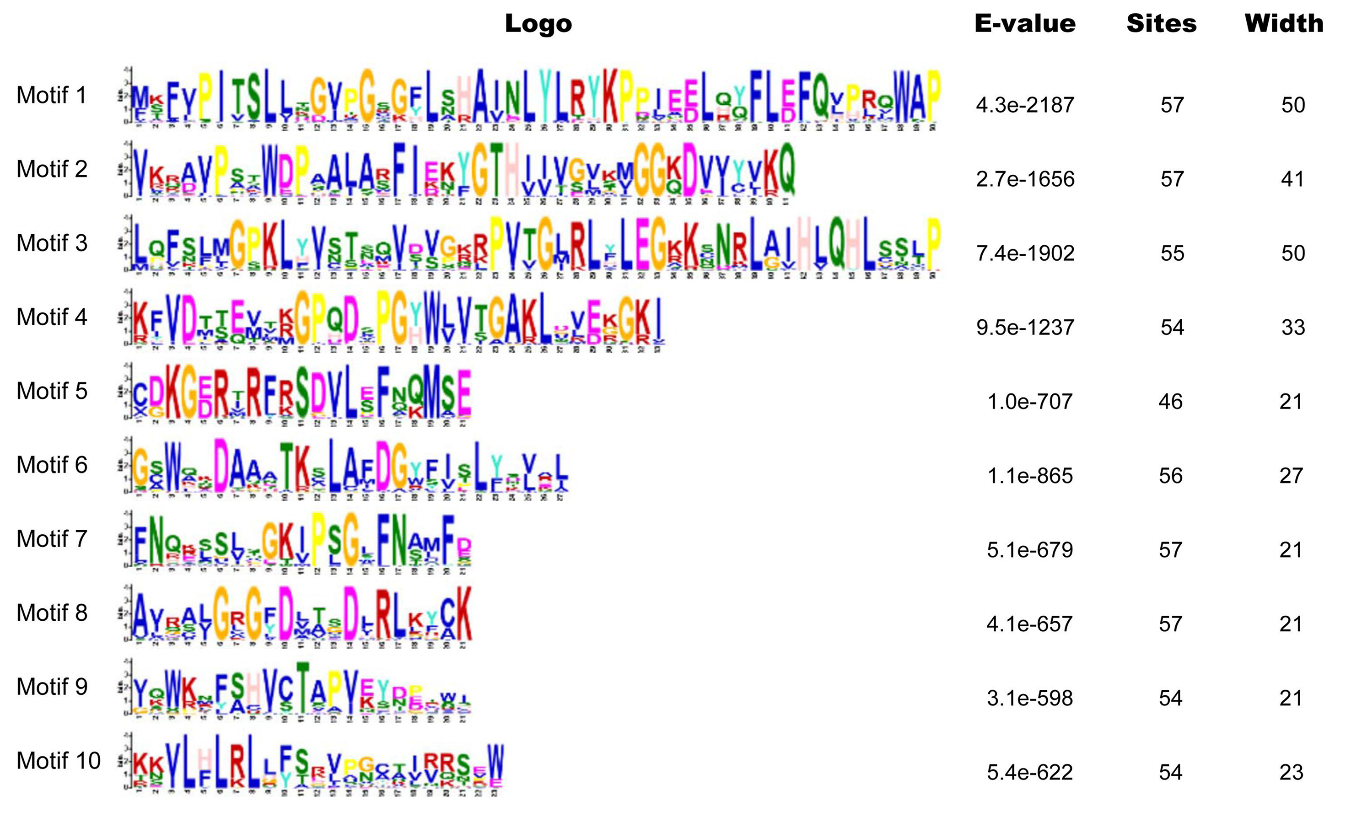


**Figure S2.** Motif sequences of the plant MACPF proteins. MACPF motif sequences were revealed by employing the MEME database. Logo height indicates the relative proportion of each amino acid letter at the given position. E-value indicates the statistical significance of the motif. Sites indicate the number of sites contributing to the construction of the motif. Width indicates the number of amino acids comprising the motif.


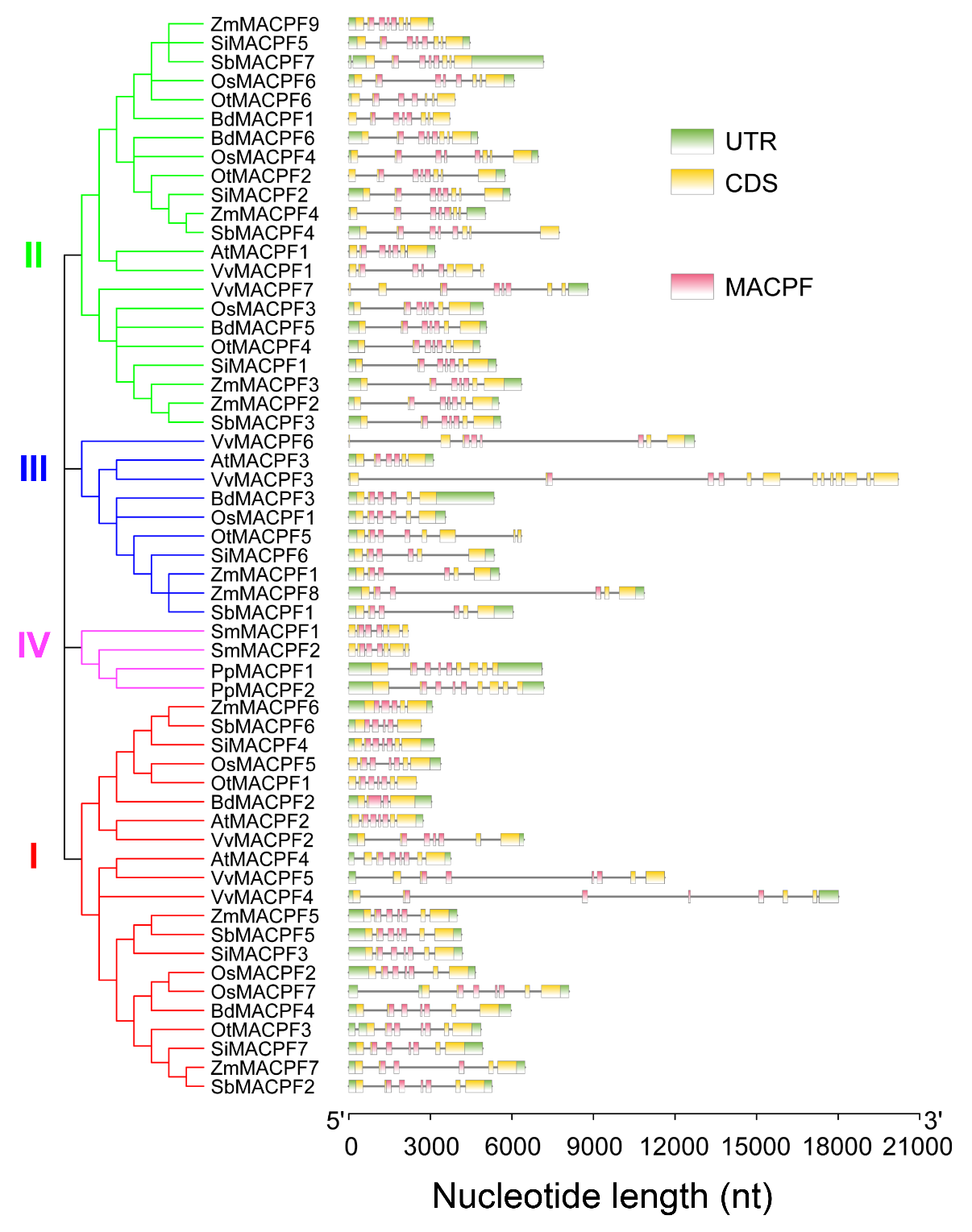


**Figure S3.** Schematic representation of predicted *cis*-regulatory elements of *MACPF* promoters. Left: phylogenetic tree of the MACPF family, redrawn from Figure 1. Right: the exon-intron structures of the *MACPF* genes, according to the GSDS website. Exons and introns are shown as filled boxes and grey lines, respectively. The fraction of the gene that encodes the MACPF domain is marked in red, with the remaining coding sequences marked in yellow. 5' and 3' untranslated regions (UTRs) are shown in green at the ends of the sequences.


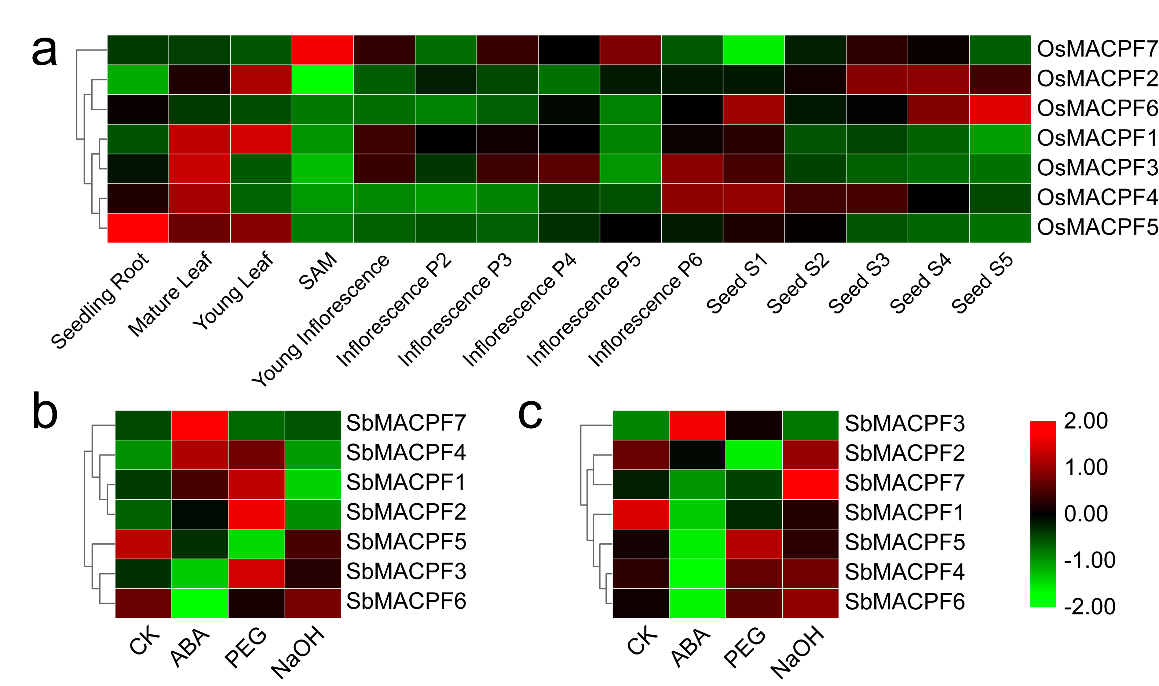


**Figure S4.** Expression patterns of *OsMACPF* genes in development and *SbMACPF* genes in response to stress. (a) Tissue-specific expression patterns of *MACPF* genes in rice, obtained from previously published data. Red and green colors represent higher and lower expression, respectively. Samples: root, leaf, SAM (shoot apical meristem) of the vegetative tissues, and different panicle and seed stages of the reproductive tissues. Expression profile of *MACPF* genes during abiotic and biotic treatments, obtained from previously published data. Expression profile of *SbMACPF* genes following ABA, PEG and NaOH treatments in shoot (b) and root (c). Heatmap was drawn in R. Red and green colors represent higher and lower expression genes in different tissues or exposed to the stresses, respectively.


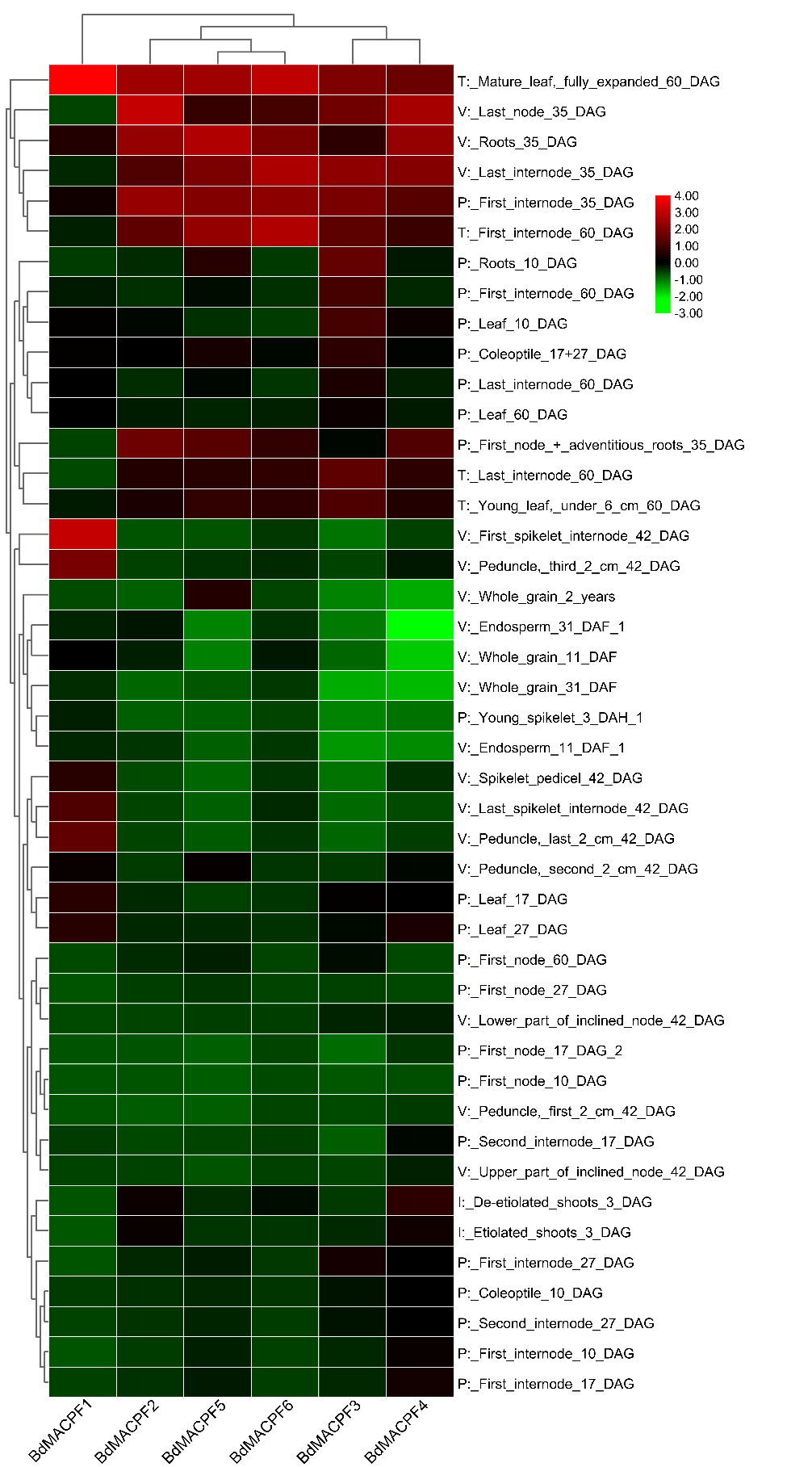


**Figure S5.** Expression patterns of *MACPF* genes in purple false brome during development. Tissue-specific expression patterns of *MACPF* genes in purple false brome, obtained from published data [50]. Heatmap was drawn in R. Red and green colors represent higher and lower expression in different tissues, respectively.

**
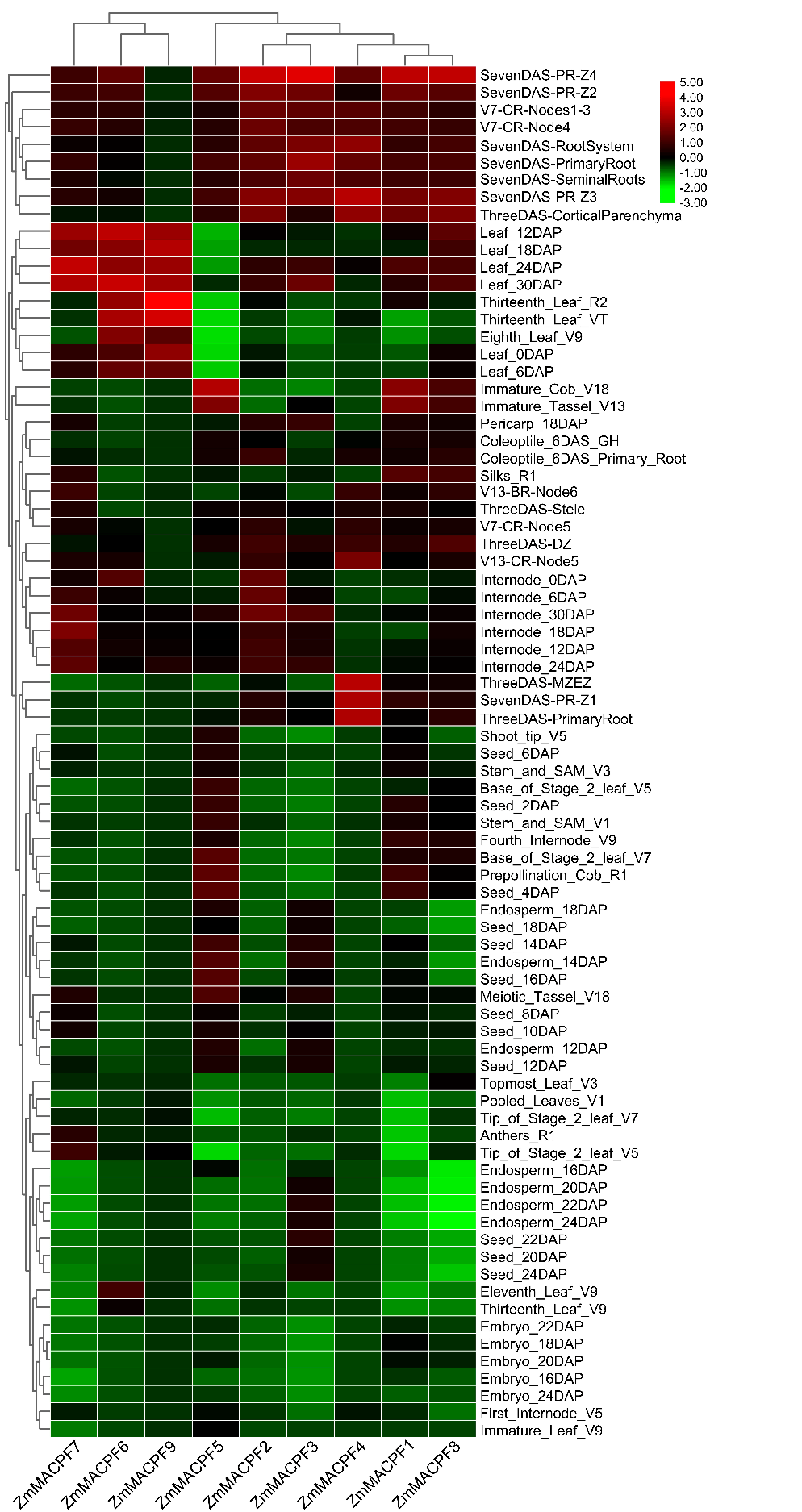
**

**Figure S6.** Expression patterns of *MACPF* genes in maize development tissues. Tissue-specific expression patterns of *MACPF* genes in maize, obtained from published data [49]. Heatmap was drawn in R. Red and green colors represent higher and lower expression in different tissues, respectively.

**Table S1. Characteristic features of 57 identified plant *MACPF* genes.**

**Table S2. KaKs ratios of segmental duplication pairs in six Poaceae species.**

**Table S3. Amino acid substitution models estimation of the Maximum Likelihood.**

**Table S4. List of *OsMACP* genes primers used for qRT-PCR.**
