# Supplementary figures and images for: Evolution and Expression of the Membrane Attack Complex and Perforin Gene Family in the Poaceae
Source: Int J Mol Sci. 2020 Aug 10;21(16):5736. doi: 10.3390/ijms21165736 (PMC7460961; doi:10.3390/ijms21165736)

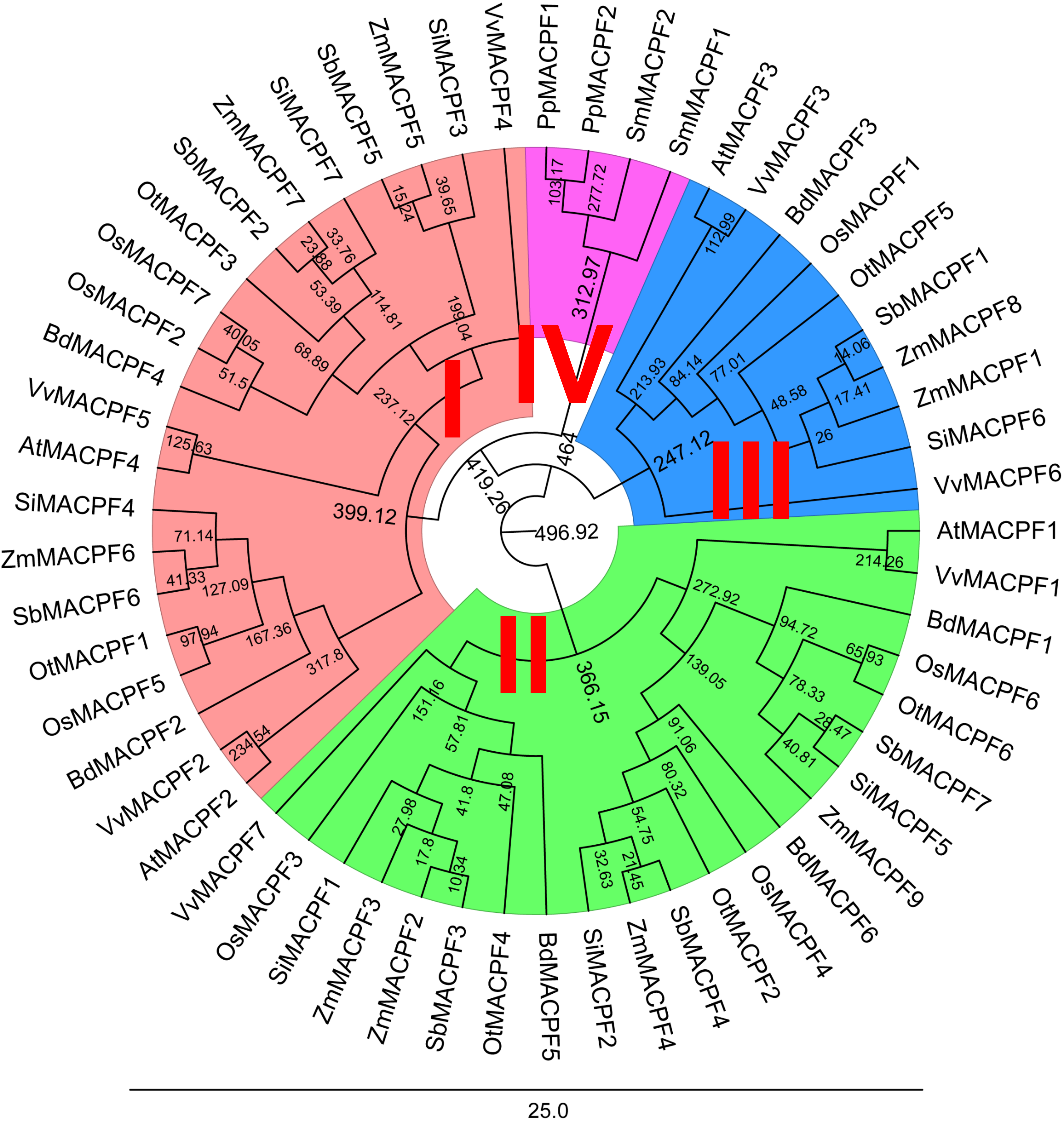

Supplement: Supplementary file 1 [file ijms-21-05736-s001.zip › ijms-884422-suppl 2/Poaceae MACPF Figure S1.tif]

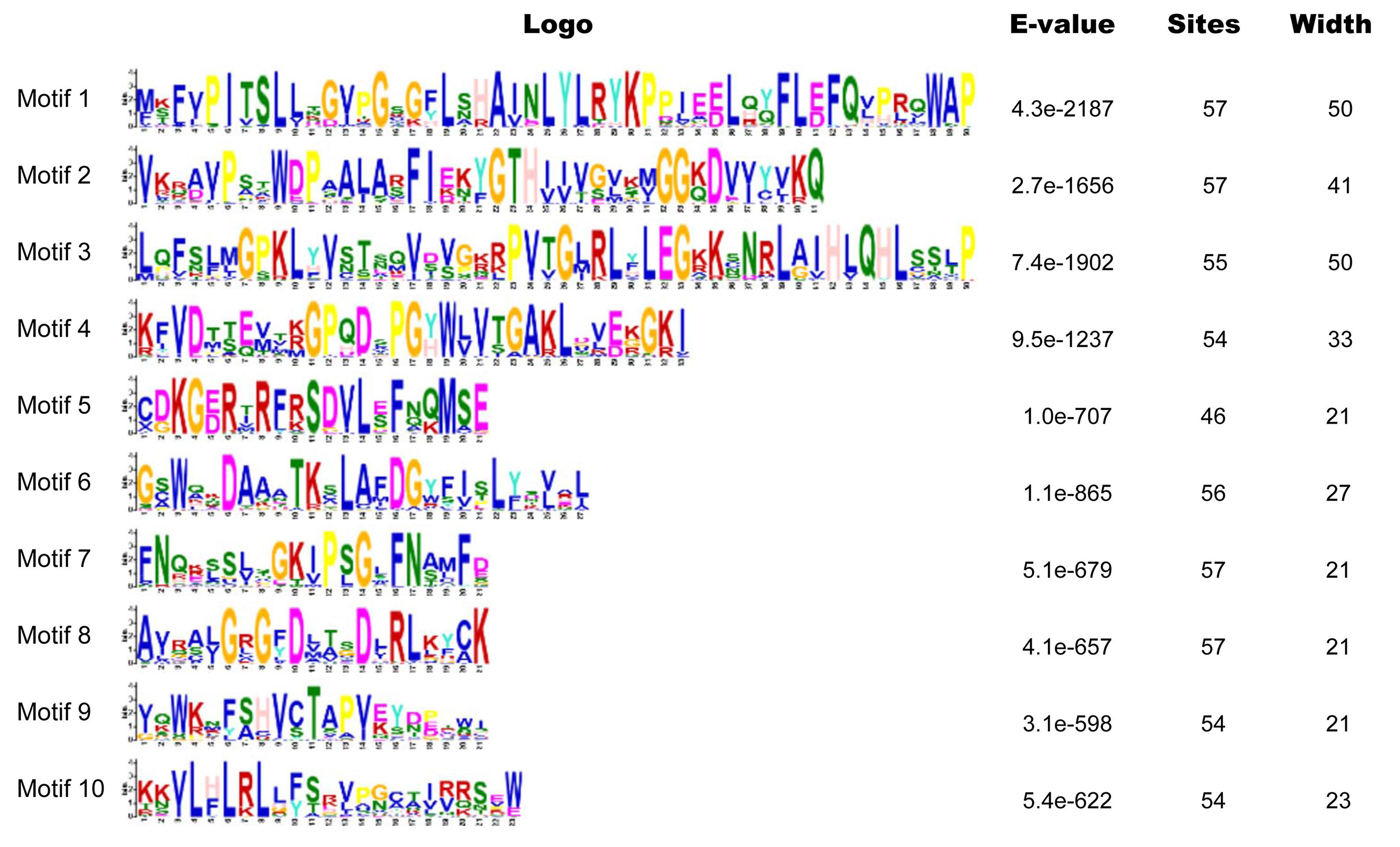

Supplement: Supplementary file 1 [file ijms-21-05736-s001.zip › ijms-884422-suppl 2/Poaceae MACPF Figure S2.tif]

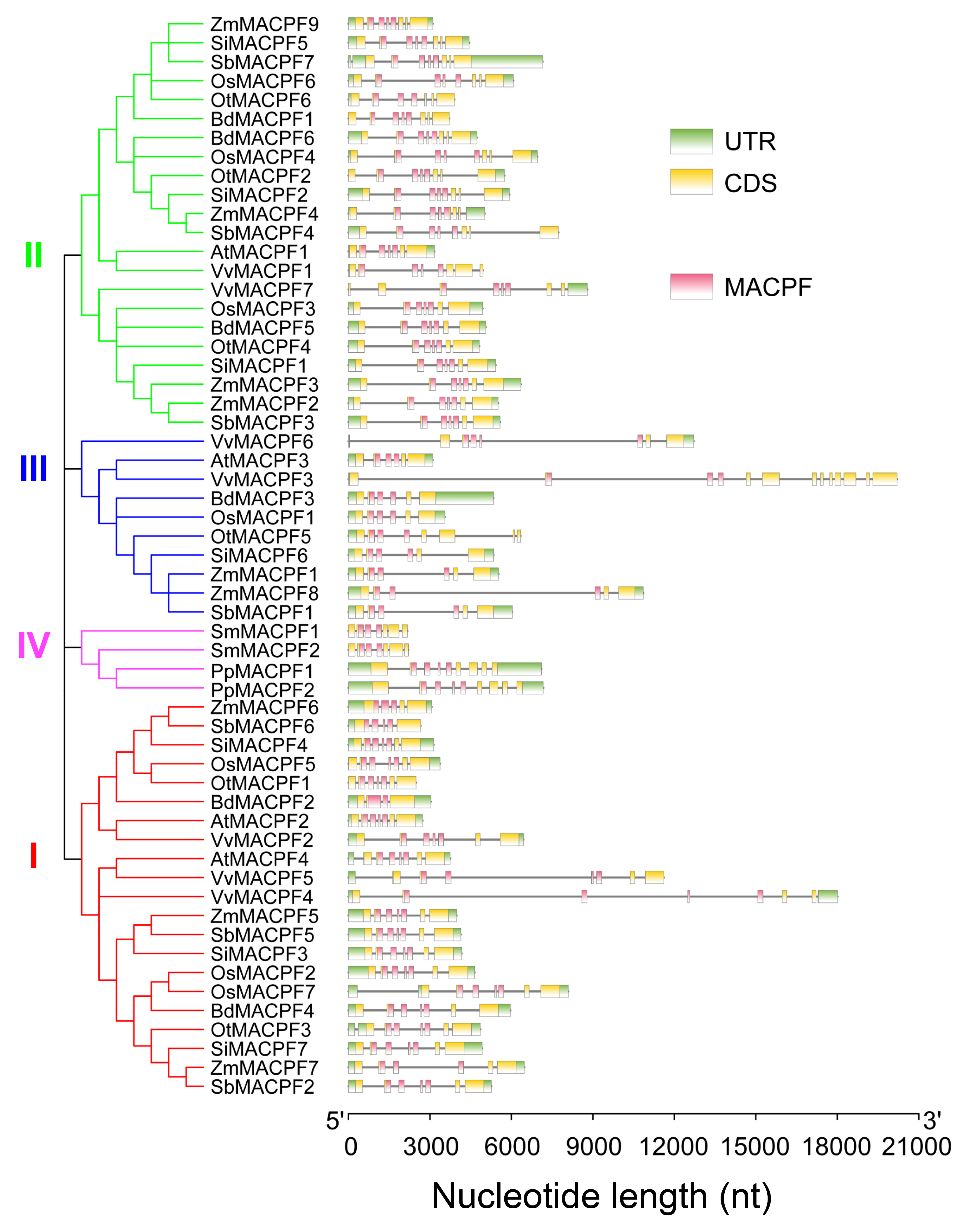

Supplement: Supplementary file 1 [file ijms-21-05736-s001.zip › ijms-884422-suppl 2/Poaceae MACPF Figure S3.tif]

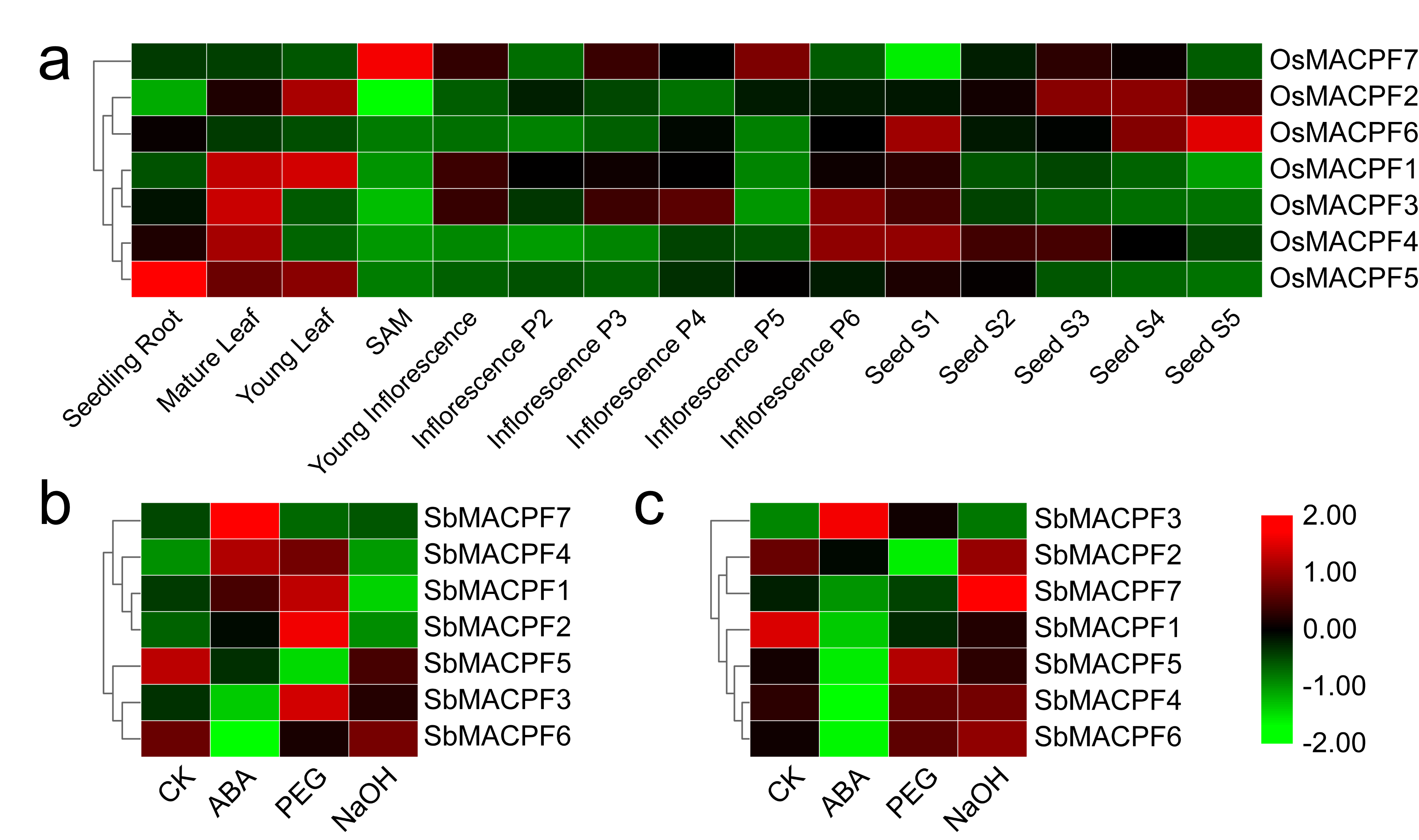

Supplement: Supplementary file 1 [file ijms-21-05736-s001.zip › ijms-884422-suppl 2/Poaceae MACPF Figure S4.tif]
